# Supplementary material for: Classification of mouse B cell types using surfaceome proteotype maps
Source: Nat Commun. 2019 Dec 16;10:5734. doi: 10.1038/s41467-019-13418-5 (PMC6915781; doi:10.1038/s41467-019-13418-5)
Supplement: Supplementary file 1 — Supplementary Information [file 41467_2019_13418_MOESM1_ESM.pdf]

Classification of Mouse B Cell Types using Surfaceome Proteotype Maps

Supplementary Information

van Oostrum and Mueller et. al.

Supplementary Figure 1

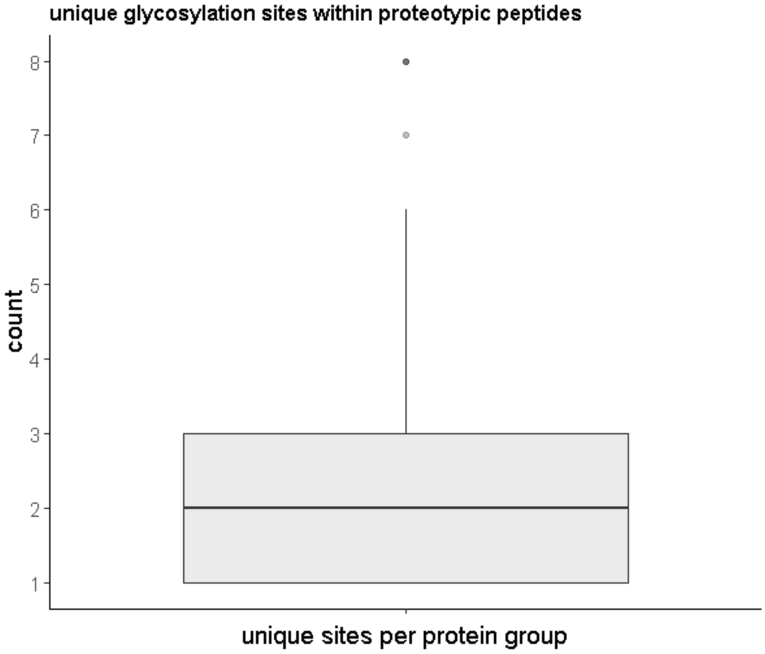

Supplementary Figure 1 | Number of identified glycosylation sites per protein group. Distribution of unique glycosylation sites within proteotypic peptides per quantified protein group. The center line of the box-plot represents the median, box limits the upper and lower quartiles, whiskers the 1.5x interquartile range and dots any outlier data points.

Supplementary Figure 2

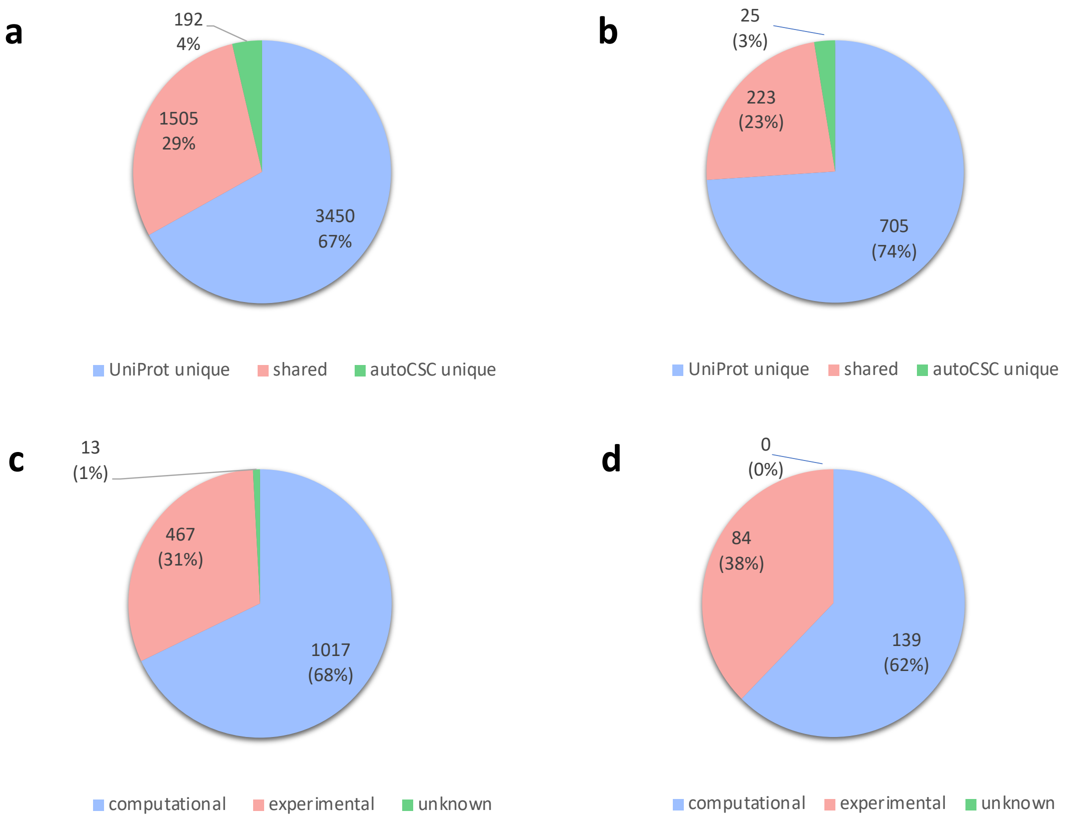

Supplementary Figure 2 | Number of glycosylation sites that are either annotated by Uniprot, identified by autoCSC or both in the surfaceome dataset of (a) 11 common human cell lines or (b) primary sorted mouse B-cells. Basis of Uniprot annotation for the glycosites shared between UniProt and the (c) surfaceome dataset of 11 human cancer cell lines or (d) developing mouse B-cells.

Supplementary Figure 3

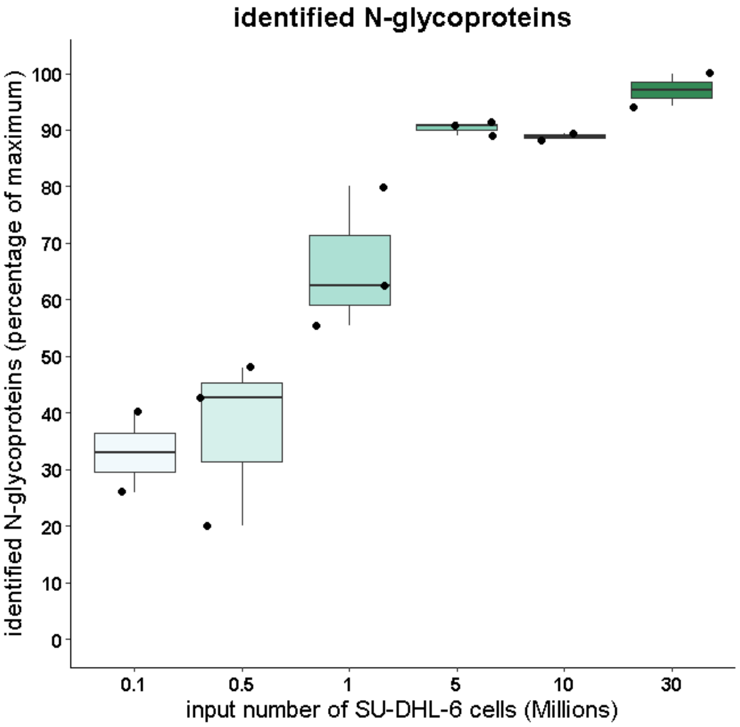

Supplementary Figure 3 | Dilution series of cell numbers used for autoCSC. Quantified N-glycopeptides using 0.1 to 30 x 10^6 SU-DHL-6 cells as input for autoCSC. The center line of box-plots represents the median, box limits the upper and lower quartiles, whiskers the 1.5x interquartile range.

Supplementary Figure 4

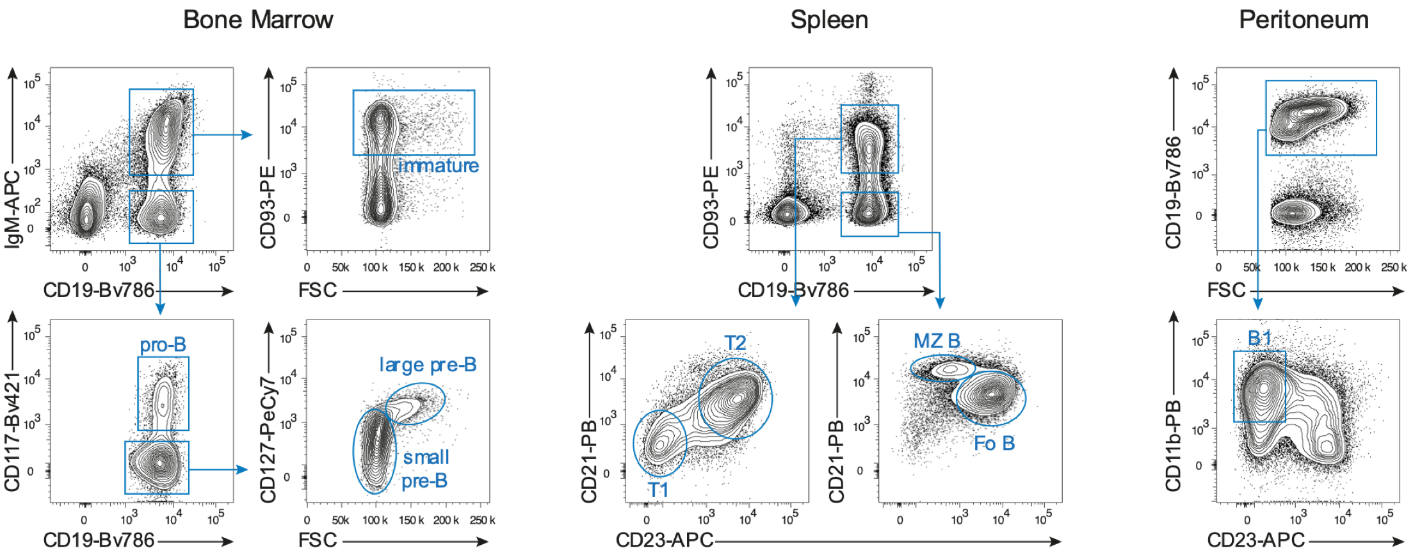

Supplementary Figure 4 | FACS strategy for mouse B cell populations. From the bone marrow: proB, $CD19^{+}IgM^{-}CD117^{+}$; large-preB, $CD19^{+}IgM^{-}CD117^{-}CD127^{+}FSC^{large}$; small-preB, $CD19^{+}IgM^{-}CD117^{-}CD127^{-}FSC^{small}$; and immature B, $CD19^{+}IgM^{+}CD93^{+}$. From the peritoneum: B1, $CD19^{+}CD23^{-}CD11b^{+}$. From the spleen: Transitional-1, $CD19^{+}CD93^{+}CD23^{-}CD21^{-}$; Transitional-2, $CD19^{+}CD93^{+}CD23^{+}CD21^{+}$; Follicular (Fo), $CD19^{+}CD93^{-}CD23^{+}CD21^{low}$; and Marginal Zone (MZ), $CD19^{+}CD93^{-}CD23^{low}CD21^{+}$.

Supplementary Figure 5

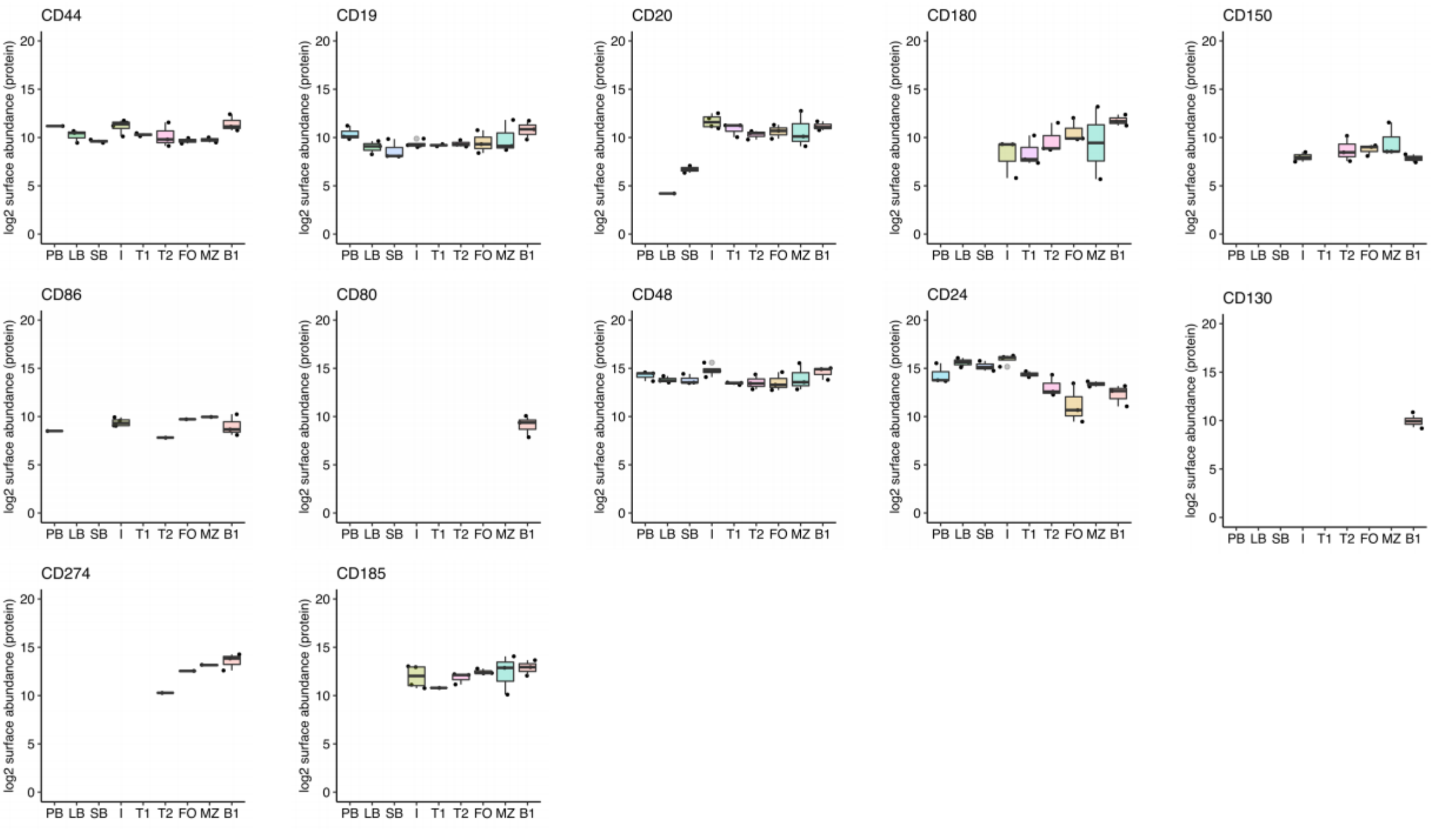

Supplementary Figure 5 | Boxplots of surface abundances across B cell populations for proteins selected for follow-up flow cytometry analysis. The center line of box-plots represents the median, box limits the upper and lower quartiles, whiskers the 1.5x interquartile range.

Supplementary Figure 6

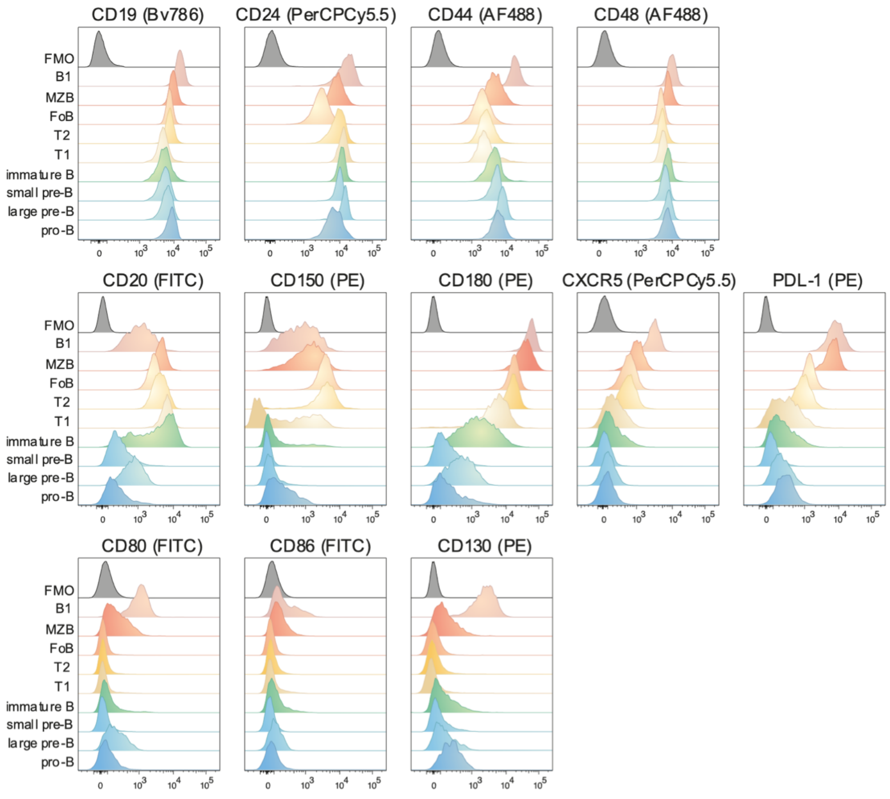

Supplementary Figure 6 | FACS plots of developing B cell populations for proteins selected from B cell surfaceomes generated by autoCSC.

Supplementary Figure 7

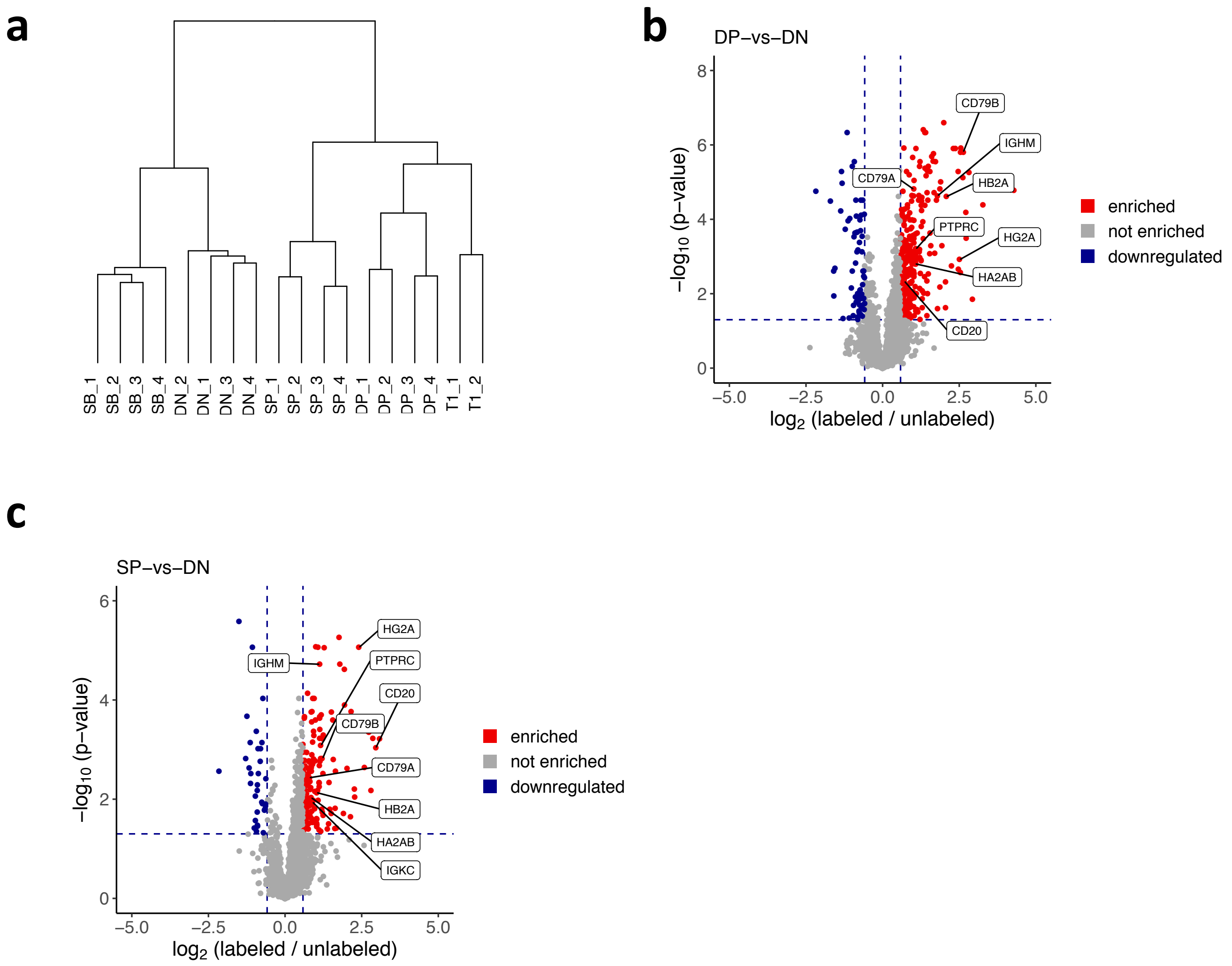

Supplementary Figure 7 | Proteotype analysis of immature B subpopulations. a Unsupervised clustering of proteotypes reflects the phenotypically defined immature B subpopulations. Cluster analysis using manhattan distance and Ward's minimum variance method. b and c Volcano plot showing statistical significance (y-axis) and surface abundance change (x-axis) comparing DP with DN or SP with DN, respectively. Significantly different proteins were determined by the threshold |fold-change| > 1.5 and adjusted p-value < 0.05 of a two-sided t-test with the appropriate degrees of freedom. Benjamini-Hochberg method was used to account for multiple testing. Selected proteins involved in B cell receptor signalling and antigen presentation are labelled. c Same for the comparison SP vs. DN.

Supplementary Figure 8

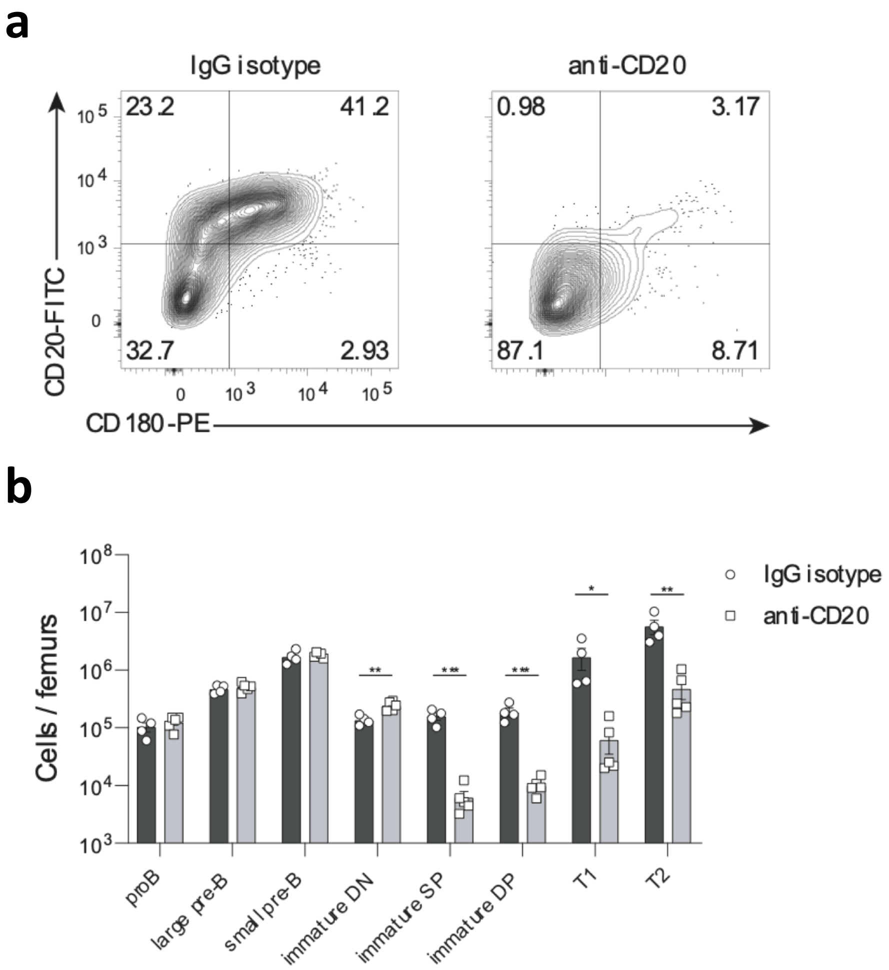

Supplementary Figure 8 | Selective sensitivity of immature B subpopulations towards depleting anti-CD20 antibodies. a FACS plots of immature B subpopulations treated with anti-CD20 antibodies or IgG isotype controls. b, Quantification of cells per femur for developing B-cell populations. Error bars indicate standard error of the mean. Statistical analysis was done with two-tailed unpaired Student's t test. One star indicates $P < 0.05$, two stars $P < 0.01$ and three stars $P < 0.001$.
