# Supplementary material for: Classification of mouse B cell types using surfaceome proteotype maps
Source: Nat Commun. 2019 Dec 16;10:5734. doi: 10.1038/s41467-019-13418-5 (PMC6915781; doi:10.1038/s41467-019-13418-5)
Supplement: Supplementary file 3 — Description of Additional Supplementary Files [file 41467_2019_13418_MOESM3_ESM.pdf]

Description of Additional Supplementary Files

File Name: Supplementary Data 1

Description: Supplementary Data 1 contains three excel spreadsheets: 1) Cell surface abundance values of autoCSC quantified N-glycoproteins on 11 commonly used cancer cell lines; 2) Cell surface abundance values of autoCSC quantified N-glycoproteins perreplicate of 11 commonly used cancer cell lines; 3) Cell surface N-glycosylation sites identified by autoCSC on 11 commonly used cancer cell lines.

File Name: Supplementary Data 2

Description: Supplementary Data 2 contains three excel spreadsheets: 1) Cell surface abundance values of autoCSC quantified N-glycoproteins on mouse primary B-cells at sequential developmental stages; 2 Cell surface abundance values of autoCSC quantified glycoproteins per replicate on mouse primary B-cells at sequential developmental stages; 3) Cell surface N-glycosylation sites identified by autoCSC on mouse primary B-cells at sequential developmental stages.

File Name: Supplementary Data 3

Description: Supplementary Data 3 contains five excel spreadsheets: 1) Abundance values resulting from proteotype analysis of immature B cell subpopulations for each sample; 2) Abundance values resulting from proteotype analysis of immature B cell subpopulations per group; 3-5) Results from significance testing in pairwise comparisons of immature B subpopulations DN, SP and DP.
