# Supplementary material for: Classification of mouse B cell types using surfaceome proteotype maps
Source: Nat Commun. 2019 Dec 16;10:5734. doi: 10.1038/s41467-019-13418-5 (PMC6915781; doi:10.1038/s41467-019-13418-5)
Supplement: Supplementary file 7 — Reporting Summary [file 41467_2019_13418_MOESM7_ESM.pdf]

nature research

Corresponding author(s): *Double-blind peer review submissions: write DBPR and your manuscript number here instead of author names.*

Last updated by author(s): *YYYY-MM-DD*

Reporting Summary

Nature Research wishes to improve the reproducibility of the work that we publish. This form provides structure for consistency and transparency in reporting. For further information on Nature Research policies, seeAuthors & Referees and theEditorial Policy Checklist .

Statistics

For all statistical analyses, confirm that the following items are present in the figure legend, table legend, main text, or Methods section.

n/a	Confirmed	
☐	☒	The exact sample size (*n*) for each experimental group/condition, given as a discrete number and unit of measurement
☐	☒	A statement on whether measurements were taken from distinct samples or whether the same sample was measured repeatedly
☐	☒	The statistical test(s) used AND whether they are one- or two-sided<br>*Only common tests should be described solely by name; describe more complex techniques in the Methods section.*
☒	☐	A description of all covariates tested
☐	☒	A description of any assumptions or corrections, such as tests of normality and adjustment for multiple comparisons
☐	☒	A full description of the statistical parameters including central tendency (e.g. means) or other basic estimates (e.g. regression coefficient) AND variation (e.g. standard deviation) or associated estimates of uncertainty (e.g. confidence intervals)
☒	☐	For null hypothesis testing, the test statistic (e.g. *F*, *t*, *r*) with confidence intervals, effect sizes, degrees of freedom and *P* value noted<br>*Give P values as exact values whenever suitable.*
☒	☐	For Bayesian analysis, information on the choice of priors and Markov chain Monte Carlo settings
☒	☐	For hierarchical and complex designs, identification of the appropriate level for tests and full reporting of outcomes
☒	☐	Estimates of effect sizes (e.g. Cohen's *d*, Pearson's *r*), indicating how they were calculated

Our web collection on statistics for biologists contains articles on many of the points above.

Software and code

Policy information about availability of computer code

Data collection	Mass spectra were acquired using a Thermo Fisher Scientific mass spectrometers operated with the vendor provided Tune and Xcalibur software. RNA sequencing data was retrieved from ImmGen database (https://www.immgen.org/). For flow cytometry a BD LSRFortessa (BD Biosciences) was used. For cell sorting, a FACSAria IIu (BD Biosciences) was used.
Data analysis	Acquired spectra containing raw files were processed with either Trans Proteomic Pipeline (TPP, v4.7 Build 201402281256, Seattle Proteome Center) using the Comet search engine or Proteome Discoverer (v.2.1, Thermo Scientific) using SEQUEST search engine. Spectronaut (v.12, Biognosys AG) or Progenesis QI (v.4.0, Nonlinear Dynamics) was used for quantitative analysis of DIA and DDA identified peptides. Bioinformatic analysis was performed in python (v.3.6) and the R statistical computing environment (v.3.4.0) using the R package MSstats (v.3.8.6). Flow cytometry data was collected using FACSDiva software and analysed using FlowJo Software.

For manuscripts utilizing custom algorithms or software that are central to the research but not yet described in published literature, software must be made available to editors/reviewers. We strongly encourage code deposition in a community repository (e.g. GitHub). See the Nature Research guidelines for submitting code & software for further information.

Data

Policy information about availability of data

All manuscripts must include a data availability statement. This statement should provide the following information, where applicable:

- Accession codes, unique identifiers, or web links for publicly available datasets
- A list of figures that have associated raw data
- A description of any restrictions on data availability

The mass spectrometry proteomics data have been deposited to the ProteomeXchange Consortium via the PRIDE partner repository with the dataset identifier PXD013627.

Field-specific reporting

Please select the one below that is the best fit for your research. If you are not sure, read the appropriate sections before making your selection.

- [x] Life sciences
- [ ] Behavioural & social sciences
- [ ] Ecological, evolutionary & environmental sciences

For a reference copy of the document with all sections, see nature.com/documents/nr-reporting-summary-flat.pdf

Life sciences study design

All studies must disclose on these points even when the disclosure is negative.

Sample size	Sample size was not predetermined by statistical methods for relative quantitative mass spectrometry experiments. However, for each protein, quantitative values were modeled based on the intensity of at least two features (peptide fragments) per proteotypic peptide using the Tukey's median polish method to ensure robust label-free quantification suitable for statistical testing with sample size at least n=3.
Data exclusions	Peptide identifications mapping to decoy or contaminant proteins or internal reference peptides were excluded from further analysis. Additionally, peptides were filtered based on MS/MS identification score to ensure a false discovery rate of < 1%. Outliers based on hierarchical clustering were removed to retain minimally three biological replicates per condition.
Replication	Automated MS-based Cell Surface Capture (autoCSC) was used to track quantitative changes of the cell surface proteotype during B-cell development. Surface expression of selected candidates was assessed and reported using antibody-based flow cytometry. Overall we found a median spearman's rho correlation value of 0.77 between both methods.
Randomization	Randomization was not applied.
Blinding	Investigators were not blinded to allocation of biological samples. The results of the mass spectrometry and flow cytometry experiments are of technical nature and not prone to a potential observer bias.

Reporting for specific materials, systems and methods

We require information from authors about some types of materials, experimental systems and methods used in many studies. Here, indicate whether each material, system or method listed is relevant to your study. If you are not sure if a list item applies to your research, read the appropriate section before selecting a response.

Materials & experimental systems

n/a	Involved in the study	
[ ]	[x]	Antibodies
[ ]	[x]	Eukaryotic cell lines
[x]	[ ]	Palaeontology
[ ]	[x]	Animals and other organisms
[x]	[ ]	Human research participants
[x]	[ ]	Clinical data

Methods

n/a	Involved in the study	
[x]	[ ]	ChIP-seq
[ ]	[x]	Flow cytometry
[x]	[ ]	MRI-based neuroimaging

Antibodies

Antibodies used	The following antibodies were used for flow cytometry (from BD Biosciences, eBioscience, BioLegend, or produced in house): anti-CD117 (2B8), anti-CD19 (1D3), anti-CD127 (SB/199), anti-IgM (M41), anti-IgD (1.19), anti-CD93 (PB493), anti-CD11b (M1.7015), anti-CD23 (B3B4), anti-CD44 (IM7), anti-CD48 (HM48-1), anti-CD24 (M1/69), anti-CD20 (SA275A11), anti-CD180 (RP/14), anti-CD150 (TC15-12F12.2), anti-CXCR5 (2G8), anti-PD-L1 (10F.9G2)), anti-CD130 (4H1B35), anti-CD80 (16-10A1), anti-CD86 (GL1), an anti-mBAFF-R (9B9)
Validation	Commercial antibodies were quality control tested by immunofluorescent staining with flow cytometric analysis by the manufacturer. Antibodies produced in house were titrated and validated using immunofluorescent staining together with a corrsponding antibody for co-staining followed by flow cytometric analysis.

Eukaryotic cell lines

Policy information about cell lines

Cell line source(s)	All cell lines were purchased from ATCC, except 16HBE14o-, which was a kind gift from Jason Mercer (MRC-Laboratory for Molecular Cell Biology, University College London)

Authentication	Previously established and commonly used cell lines were selected for comprehensive cell surface proteotype profiling using autoCSC reveling cell line specific signatures. No further authentication was applied.
Mycoplasma contamination	In the context of this study, cell lines were not specifically tested for Mycoplasma contamination.
Commonly misidentified lines (See ICLAC register)	No commonly misidentified cell lines were used.

Animals and other organisms

Policy information about studies involving animals; ARRIVE guidelines recommended for reporting animal research

Laboratory animals	Species: Mus Musculus; Strain: C57BL/6; Age: 5-7 weeks; Gender: Male and females
Wild animals	*Provide details on animals observed in or captured in the field; report species, sex and age where possible. Describe how animals were caught and transported and what happened to captive animals after the study (if killed, explain why and describe method; if released, say where and when) OR state that the study did not involve wild animals.*
Field-collected samples	*For laboratory work with field-collected samples, describe all relevant parameters such as housing, maintenance, temperature, photoperiod and end-of-experiment protocol OR state that the study did not involve samples collected from the field.*
Ethics oversight	All animal experiments were carried out under institutional guidelines (authorization number 1888 from canton Basel-Stadt veterinary office)

Note that full information on the approval of the study protocol must also be provided in the manuscript.

Flow Cytometry

Plots

Confirm that:

- [x] The axis labels state the marker and fluorochrome used (e.g. CD4-FITC).
- [x] The axis scales are clearly visible. Include numbers along axes only for bottom left plot of group (a 'group' is an analysis of identical markers).
- [x] All plots are contour plots with outliers or pseudocolor plots.
- [x] A numerical value for number of cells or percentage (with statistics) is provided.

Methodology

Sample preparation	Cells were flushed from femurs and tibias of the two hind legs and from the peritoneum of the mice or single-cell suspensions of spleen cells were made. Staining was performed in PBS containing 0.5% BSA and 5 mM EDTA.
Instrument	For flow cytometry a BD LSRFortessa (BD Biosciences) was used. For cell sorting, a FACSAria IIu (BD Biosciences) was used.
Software	Data were collected using FACSDiva software and analysed using FlowJo Software.
Cell population abundance	About 1mio cells were sorted of each population. Post-sort analysis confirmed purity >98% in all cases.
Gating strategy	FSC-A and SSC-A were used to gate on lymphocytes. SSC-W and SSC-H were used to exclude duplets. For analysis PI was used to exclude dead cells. CD19 was used in all cases to gate on B-cells. Precurser, immature and recirculating B cells in the bone marrow were splitted up using the markers IgM, CD117, CD127, and CD93. Transitional and mature B cell populations in the spleen were defined by their expression of CD93, CD21, and CD23. B1 B cells in the peritoneum were defined using CD11b and CD23.

- [x] Tick this box to confirm that a figure exemplifying the gating strategy is provided in the Supplementary Information.
